# Lamin B1 overexpression increases nuclear rigidity in autosomal dominant leukodystrophy fibroblasts

**DOI:** 10.1096/fj.13-247635

**Published:** 2014-09

**Authors:** Denise Ferrera, Claudio Canale, Roberto Marotta, Nadia Mazzaro, Marta Gritti, Michele Mazzanti, Sabina Capellari, Pietro Cortelli, Laura Gasparini

**Affiliations:** * Department of Neuroscience and Brain Technologies,; † Department of Nanophysics, and; ‡ Department of Nanochemistry, Istituto Italiano di Tecnologia, Genoa, Italy;; § Department of Biosciences, University of Milano, Milan, Italy;; ‖ Istituto di Ricovero e Cura a Carattere Scientifico (IRCCS) Istituto delle Scienze Neurologiche di Bologna, Clinica Neurologica, Ospedale Bellaria, Bologna, Italy; and; ¶ Department of Biomedical and Neuromotor Sciences, University of Bologna, Bologna, Italy

**Keywords:** ADLD, nuclear lamina, atomic force microscopy, human fibroblasts, nucleus

## Abstract

The architecture and structural mechanics of the cell nucleus are defined by the nuclear lamina, which is formed by A- and B-type lamins. Recently, gene duplication and protein overexpression of lamin B1 (LB1) have been reported in pedigrees with autosomal dominant leukodystrophy (ADLD). However, how the overexpression of LB1 affects nuclear mechanics and function and how it may result in pathology remain unexplored. Here, we report that in primary human skin fibroblasts derived from ADLD patients, LB1, but not other lamins, is overexpressed at the nuclear lamina and specifically enhances nuclear stiffness. Transient transfection of LB1 in HEK293 and neuronal N2a cells mimics the mechanical phenotype of ADLD nuclei. Notably, in ADLD fibroblasts, reducing LB1 protein levels by shRNA knockdown restores elasticity values to those indistinguishable from control fibroblasts. Moreover, isolated nuclei from ADLD fibroblasts display a reduced nuclear ion channel open probability on voltage-step application, suggesting that biophysical changes induced by LB1 overexpression may alter nuclear signaling cascades in somatic cells. Overall, the overexpression of LB1 in ADLD cells alters nuclear mechanics and is linked to changes in nuclear signaling, which could help explain the pathogenesis of this disease.—Ferrera, D., Canale, C., Marotta, R., Mazzaro, N., Gritti, M., Mazzanti, M., Capellari, S., Cortelli, P., Gasparini, L. Lamin B1 overexpression increases nuclear rigidity in autosomal dominant leukodystrophy fibroblasts.

The nuclear lamina is the filamentous meshwork that underlies the inner nuclear membrane of eukaryotic cells. It has unique elastic and compressibility properties and appears to act as a molecular shock absorber (1, 2), with behavior that is typical of a solid-elastic shell. Lamin B1 (LB1) is a major component of the nuclear lamina and, together with other lamins (*e.g.*, lamin A/C), plays a role in the structure and function of the nucleus (3). Over the past few decades, mutations in lamin A (LA) and lamin-associated proteins have been shown to cause various human diseases. These diseases are termed laminopathies and include Emery-Dreifuss and limb-girdle muscular dystrophies, dilated cardiomyopathy, Dunningan-type familial partial lipodystrophy, Charcot-Marie tooth disorder type 2, and Hutchison-Gilford progeria syndrome (4).

The role of LB1 in pathological conditions was discovered only recently, when the duplication of the gene encoding this protein was associated with adult-onset autosomal dominant leukodystrophy (ADLD) (5–7). ADLD is the first identified laminopathy that affects the central nervous system and is characterized by widespread cerebral demyelination (8–12), as well as autonomic and pyramidal symptoms (13). Pathogenesis may be linked to altered mechanical properties of the nucleus. Changes in other lamins alter the nuclear lamina and affect the structural integrity of the nucleus and its sensitivity to mechanical stress. For example, lamins A and C (LA/C) play a key role in defining the nuclear lamina's elastic properties: LA/C-deficient cells have increased numbers of misshapen nuclei and reduced nuclear stiffness (14). Moreover, when wild-type (15) or mutant LA is overexpressed (16), the stiffness of Xenopus oocyte nuclei increases proportionally to the LA concentration.

Unlike LA/C, the contribution of LB1 to the elastic properties of the nucleus is unclear. In LB1-deficient mouse embryonic fibroblasts (MEFs), although nuclei respond normally to biaxial strain, there are severe abnormalities in nuclear morphology (14). However, it remains unclear whether the pathological overexpression of LB1 that is associated with ADLD alters nuclear mechanics and function in somatic cells.

To address this central question, we examined how LB1 impacts nuclear rigidity and function, using primary cultures of human skin fibroblasts derived from patients with ADLD bearing a duplication of the LB1 gene. Overexpressed LB1 is primarily localized to the nuclear lamina and results in increased nuclear stiffness. In HEK293 and neuronal N2a cells, the overexpression of LB1 significantly increases nuclear rigidity, mimicking the mechanical phenotype of ADLD nuclei. When LB1 expression is reduced in ADLD fibroblasts *via* LB1 shRNA, nuclear stiffness reverts to levels indistinguishable from those of control (CTR) fibroblasts. Further, in nuclei isolated from ADLD fibroblasts, while single channel conductance is preserved, the open probability of the ion channel is reduced with increasing voltage steps. This suggests that, in somatic cells, LB1 overexpression causes biophysical changes in the nuclear lamina that alter nuclear signaling, possibly leading to nuclear dysfunction in ADLD.

## MATERIALS AND METHODS

### Patients with ADLD and CTR subjects

Skin biopsies were obtained from 2 affected ADLD patients carrying *LB1* gene duplications (17), 6 noncarrier siblings, and 6 healthy volunteers who were age matched with patients at the Neurological Clinic of the University of Bologna. The study has been approved by the AUSL Bologna Ethical Committee, and informed consent was obtained from all of participants. The clinical features of the patients were as follows. At 48 yr of age, the index patient reported micturition urgency and impotence followed by progressive asthenia, objective vertigo, orthostatic syncope, constipation, and motor difficulties of the left lower limb with poor balance. Neurological examination revealed spinal and cerebellar signs and a severe symptomatic orthostatic hypotension. His 57 yr old sister was examined following a 7 yr history of slowly progressive clumsy gait and urinary urgency. Neurological examination revealed cerebellar signs. Cardiovascular reflexes revealed borderline results. In both patients, the brain MRI revealed a diffuse T2 hyperintensity of the cerebral white matter with a predominant frontoparietal distribution.

The CTR subjects (mean age±sd, 52.17±4.45) and noncarrier siblings (mean age±sd, 36.83±2.86) did not present relevant pathologies.

### Primary cultures of human skin fibroblasts

The primary cultures of human skin fibroblasts were established as described previously (18). For the atomic force microscopy (AFM) studies, the fibroblasts were analyzed at 4 d of proliferation in complete medium (proliferating cells) or following serum deprivation for 24 h (quiescent cells). In selected experiments, the cells were reexposed to complete medium following serum deprivation for 1–5 d to test the reversibility of the quiescent state.

### Proliferation ssays

The fibroblasts were plated at 300,000 cells per 75 cm^2^ flasks. The cells were harvested weekly by trypsinization and counted using a hemocytometer. The population doubling (PD) was calculated using the following equation: PD = log(*N*_h_/*N*_s_)log2, where *N*_h_ is the number of harvested cells and *N*_s_ is the number of seeded cells.

For the bromodeoxyuridine (BrdU) incorporation assays, the cells were plated onto poly-l-lysine-coated glass coverslips, exposed to 10 μM BrdU for 6 h, and fixed for 30 min in 4% paraformaldehyde (PFA) in PBS (pH 7.4). The BrdU immunocytochemistry was performed as described previously, with minor modifications (19). The nuclei were counterstained with Hoechst-33342 and imaged using a Nikon Eclypse 80i upright microscope that was equipped with a CFI planAPO VC 20 times] A.N. 0.80 d.l. 1 mm objective lens and a digital DS-5 Mc CCD camera (Nikon). BrdU-positive nuclei were counted and expressed as a percentage of total nuclei.

### MEF and HEK293 cell cultures

LB-null (LB1Δ/Δ) mice (20) were obtained from the Mutant Mouse Regional Resource Center (MMRRCl University of California, Davis, CA, USA). Animal health and comfort were veterinary controlled. The mice were housed in filtered cages in a temperature-controlled room with a 12:12 h dark/light cycle with *ad libitum* access to water and food. All of the animal experiments were performed in accordance with the European Community Council Directive dated November 24, 1986 (86/609/EEC) and were approved by the Italian Ministry of Health and by the IIT Ethical Committee. MEFs were isolated from wild-type LB+/+ homozygous and LB1Δ/Δ embryos at 13.5 d of gestation. The cells were isolated by digestion in 0.25% Trypsin-EDTA (Invitrogen, Carlsbad, CA, USA) and 40 μg/ml DNase I at 37°C for 1 h and mechanical dissociation. The fibroblasts were cultured at 37°C in a humidified incubator with 5% CO_2_. The offspring genotypes were determined using primers specific for the wild-type *lb1* allele (forward, 5′-TCCGTGTCGTGTGGTAGGAGG-3′; and reverse, 5′-GCAGGAGGGTTGGGAAAGCC-3′) and for the mutant allele carrying the gene-trap insertion (forward, as above; reverse, 5′-CACTCCAACCTCCGCAAACTC-3′). The genotype was confirmed by Western blot analysis of fibroblast protein extracts.

HEK293 cells were cultured in DMEM with 4.5 g/l glucose that was supplemented with 10% FCS and antibiotics. Neuronal N2a cells were cultured as described previously (21).

### Plasmids, transfection, and cell sorting

The pCAGGS-LB1-IRES-EGFP plasmid was obtained by cloning human LB1 cDNA (NM_005573.3) into the pCAGGS-IRES-EGFP vector (22), kindly donated by Dr. L. Cancedda (Istituto Italiano di Tecnologia, Genova, Italy). LB1 shRNA and scrambled shRNA plasmids containing the enhanced green fluorescent protein (EGFP) reporter were a kind gift from Dr. R. Goldman (Northwestern University, Chicago, IL, USA). The construction of the shRNA expression plasmids was described elsewhere (23, 24). The target sequences for LB1 silencing were as follows: *LB1* T3, 5′-CGAGCATCCTCAAGTCGTA-3′ (human LB1); *LB1* T4, 5′-GAATCAGAGGCGAGTAGTA-3′ (human LB1); and Neg-ctrl, 5′-ATGTACTGCGCGTGGAGA-3′ (negative control, scrambled). The loop sequence is 5′-TTCAAGAGA-3′.

Lipofection (Lipofectamine; Invitrogen) or the Amaxa Nucleofector device (Lonza, Basel, Switzerland) was used to transfect subconfluent HEK293 cells and ADLD human skin fibroblasts, respectively (19). Forty-eight to 72 h later, the transfected cells were sorted by EGFP expression in FACSFlow buffer using the BD FACS Aria II Cell Sorter [fluorescence-activated cell sorting (FACS); Becton Dickinson, Franklin Lakes, NJ, USA] at a pressure of 3100 mbar and an aspiration rate of 10 μl/min. The nuclei were extracted from EGFP-positive cells and were analyzed using AFM.

### Flow cytometry analysis

For the DNA content analysis, the fibroblasts were detached by trypsinization and incubated on ice for 10 min with 50 μg/ml propidium iodide in 0.1% sodium citrate, 0.1 mg/ml RNase A, and 0.1% Triton X-100. The cells were immediately analyzed using flow cytometry using the BD FACS Aria II Cell Sorter with FACSDiva software.

### Antibodies and reagents

The following primary antibodies were used: mouse monoclonal antibodies against LB1 (Zymed, San Francisco, CA, USA); LB2 (Abcam, Cambridge, MA, USA); LA/C (Millipore, Billerica, MA, USA); rabbit polyclonal antiserum against LB1 (Abcam); LA/C (Santa Cruz Biotechnology, Santa Cruz, CA, USA), trimethyl histone H3 (Lys27) (Millipore); γ-tubulin, β-tubulin and β-actin (Sigma, St. Louis, MO, USA); and BU1/75 (ICR1) rat monoclonal anti-BrdU antibody (Abcam). To analyze the localization of nuclear components, the following primary antibodies were used: anti-Nup153 (Abcam), anti-nuclear pore complex (mab 414; Covance, Princeton, NJ, USA), anti-sc-35 (Sigma), anti-LAP2β (BD Biosciences, San Diego, CA, USA), and anti-fibrillarin (Cytoskeleton Denver, CO, USA) mouse monoclonal antibodies, as well as antiactivated RNA Polymerase II monoclonal IgM (Covance).

For the Western blot analysis, horseradish-peroxidase-conjugated secondary antibodies (Bio-Rad Laboratories, Hercules, CA, USA) were used for detection. For the immunofluorescence analyses, Alexa fluorophore-conjugated anti-mouse, anti-rabbit, or anti-rat antibodies from Invitrogen were used.

Unless otherwise specified, the general reagents and chemicals were from Sigma, and the reagents for the cell cultures were from Invitrogen.

### Western blot analysis

Confluent fibroblast monolayers were lysed by boiling for 5 min in 1% SDS, 1 mM EDTA, 5 mM HEPES (pH 7.4), and protease inhibitors. The cells were then sonicated and spun at 20,000 *g* for 20 min at room temperature (RT). Equal amounts of proteins were separated on NuPage 10% bis-tris polyacrylamide gels (Invitrogen) and were analyzed by Western blotting, as described previously (25), using the antibodies listed above. Densitometric analysis was performed using the U.S. National Institutes of Health (NIH) ImageJ program (26). The protein levels were expressed as a ratio with respect to β- or γ-tubulin levels and normalized to CTR protein expression.

### Salt extraction

The salt extraction of nuclei was performed as previously reported (27), with minor modifications. Briefly, the nuclei were extracted from serum-deprived confluent monolayers by osmotic lysis and were washed in 10 mM Tris HCl pH 7.4, 2 mM MgCl_2_, and 0.1% Triton X-100. Sequential extraction was then performed with 50 mM and 500 mM NaCl. At each step, the nuclei were incubated for 5 min in extraction buffer on ice and then spun at 700 *g* for 5 min at 4°C. The final pellet that contained the insoluble fraction was lysed in 1% SDS, 1 mM EDTA, 5 mM HEPES (pH 7.4), and protease inhibitors by boiling and sonication. The fractions were analyzed by Western blot, as described above.

### Immunofluorescence

The fibroblasts were plated onto poly-L-lysine-coated coverslips, serum-deprived for 24 h, fixed in 4% PFA, and immunolabeled as described previously (25, 28).

To count the nuclei with irregular morphology, the samples were immunostained using rabbit polyclonal anti-LB1 or goat anti-LB and counterstained with Hoechst-33342. The confocal optical sectioning was performed at RT using a Leica TCS SP5 AOBS TANDEM inverted confocal microscope that was equipped with a HCX PL APO 40 ×1.25 Oil and a HCX PL APO λ blue 63 ×1.4 NA Oil objective lenses. The nuclei were scored as normal (oval or spherical shape) or as misshapen (irregular contour, the presence of blebs and membrane invaginations) by an operator who was blind to the nature of the samples. The count of RNA polymerase II-positive nuclei was performed on confocal images of blind-coded samples immunostained for RNA polymerase II by an experienced researcher. To quantify protein levels, z-stack confocal images of ADLD and CTR nuclei were acquired alongside in the same confocal session using fixed laser and scanning settings. With the use of maximal projection confocal images, nuclei were selected and the protein immunoreactive area was measured using the Leica LAS and the NIH ImageJ softwares (26). Data are normalized on the area covered by DNA staining with the Hoechst-33342 dye.

### Immunoelectron microscopy

CTR and ADLD fibroblasts were fixed for 1 h at RT in 0.2% glutaraldehyde and 2% paraformaldehyde in culture medium and processed for cryosectioning, as described previously (29). Briefly, following fixation, the cells were repeatedly washed in 0.15% glycine in PBS, resuspended in 12% gelatin, and cryoprotected overnight at 4°C in 2.3 M sucrose. The sections were mounted on cryo-pins and snap-frozen in liquid nitrogen. Ultrathin 72 nm thick cryosections were cut at −120°C using a Cryo Immuno diamond knife (Diatome) on a Leica EM UC6 cryoultramicrotome and placed on Formvar nickel-coated grids. For the immunostaining, the sections were blocked in 1% BSA in PBS and labeled with rabbit polyclonal anti-LB1 antibody (1: 50). The sections were then stained with a secondary anti-rabbit antibody conjugated to 10-nm colloidal gold (GAR Aurion, Delta Microscopie). The immunodecorated sections were stained with 2% uranyl acetate and embedded in a 9/1 mixture of methylcellulose/saturated uranyl acetate. Transmission electron microscopy images were collected using a Jeol JEM 1011 microscope operating at 100 KV and recorded with an 11 Mp Gatan Orius SC100 Charge-Coupled Device camera. The LB1 levels both at the nuclear lamina (within 30 nm from the inner nuclear membrane) and in the nucleoplasm were evaluated by counting the number of gold particles on a total of 88 randomly sampled micrographs (43 from CTR and 45 from ADLD fibroblasts), corresponding to a total nuclear area >130 μm^2^.

### AFM analysis

To avoid confounding effects of cytoskeletal components and force dissipation by cytosolic factors, we performed AFM indentation on isolated nuclei that were extracted by osmotic lysis of cells with 0.56% KCl for 30 min at RT and spun at 350 g for 5 min at RT. The nuclei were then resuspended in PBS and plated onto a poly-L-lysine-coated Petri dish by incubation for 1 h at RT. In CTR experiments, we performed AFM indentation on isolated CTR nuclei placed in PBS or in a buffer (Cyt) mimicking cytosolic ionic content containing 10 mM HEPES, 150 mM NaCl, 3 mM KCl, 22 mM sucrose, 10 mM glucose, 1 mM MgCl_2_, and 2 mM CaCl_2_ at pH 7.4 (30). Stiffness values were similar in the 2 experimental conditions (PBS, 1.71±0.20 mN/m, *n*=11; Cyt, 1.53±0.14, *n*=12; *P*=0.46, Student's *t* test). Therefore, PBS was used for AFM indentation on isolated nuclei throughout the study.

In selected experiments, we also carried out AFM indentation on adherent quiescent living cells. Fibroblasts or N2a cells were plated onto 40 mm petri dishes (TPP Techno Plastic Products, Switzerland), serum deprived and/or transfected as described above. Throughout AFM indentation measurements, cells were maintained at 37°C in a live imaging buffer containing 10 mM HEPES, 150 mM NaCl, 3 mM KCl, 22 mM sucrose, 10 mM glucose, 1 mM MgCl_2_, and 2 mM CaCl_2_ at pH 7.4 (31).

AFM indentation (32–34) was performed using the Nanowizard II AFM (JPK Instruments, Berlin, Germany) that was mounted on an Axio Observer D1 inverted optical microscope (Carl Zeiss, Oberkochen, Germany). The acquisition of force curve maps required ∼5 min per nucleus or cell. On average, 6–9 maps were acquired in each experimental session. Nuclear elasticity was probed using spherical polystyrene beads (Ø 4 μm; Polysciences, Inc., Warrington, PA USA) that were mounted on silicon tipless cantilevers TL1 (Nanosensors, Neuchatel, Switzerland) with nominal spring constant of 0.03 N/m. The actual spring constant of each cantilever was determined using the *in situ* thermal noise method (35). The maximum force applied to the sample was 1 nN. The velocity of the piezo-scanner was maintained at a constant 3 μm/s. The force curves were corrected for the bending of the cantilever (36) to calculate the tip-sample separation and to build force *vs.* indentation (F-I) curves (Supplemental Fig. S1). Nuclear stiffness was computed as described (15) by calculating the linear fit of the F-I curve between 2 specific force values (*i.e.*, F_o_=250 pN and F_1_=550 pN), corresponding to indentations of ≤1 μm. The force curves were analyzed using a custom MATLAB routine to allow the batch processing of a set of curves according to the following formula:

$$Stiffness\;\left(mN\;/m\right)=-\frac{Y_{1}-Y_{0}}{X_{1}-X_{0}}$$

where *X*_1_ and *X*_0_ are the tip-sample separation in μm and *Y*_1_ and *Y*_0_ are the deflection in nN at forces of F_1_ and F_0_, respectively (Supplemental Fig. S1). A total of 593 nuclei were analyzed. Eight-by-eight curve point force indentation maps (64 curves/nucleus) covering an area of 16 μm^2^ were acquired in the center of each nucleus using the DirectOverlay routine of the AFM acquisition software (JPK Instruments). Calculated stiffness values were typically comprised between 0.05 and 10 mN/m, with only a minor proportion (<5%) exceeding these limits. Nuclear integrity following AFM analysis was confirmed using propidium iodide exclusion and map repetition on the same nucleus (Supplemental Fig. S1).

The Young's elastic modulus (E) was calculated as described previously (37). Briefly, E was calculated by converting the force-displacement (F-D) curves into F-I curves and fitting them with the Hertz model, taking the Poisson ratio of the nucleus to be 0.5, which is typical for soft biological materials (33). The Hertz contact mechanics model can be directly applied to examine thin samples when the indentation depth is ≤10% of the sample thickness (37, 38). Therefore, to determine the correct indentation range, we reconstructed sample topography (Supplemental Fig. S2*A*) and calculated E for different indentation depths (Supplemental Fig. S2*B-D*). When the indenter contacts the nuclear surface, E rose sharply up to plateau values, which represent the Young's elastic modulus of the nucleus. Further increases in indentation (*i.e.*, indentation higher than 1.2 ± 0.2 μm; Supplemental Fig. S2*B*) caused an additional increase in E due to mixed contributions from both the nucleus and the substrate. Consistent with previous findings (37), comparable distributions of E were obtained when indentations ≤1 μm were used (Supplemental Fig. S2*C*). However, the E-frequency distribution shifted to higher values when the indentation depth increased to 1.5 μm (Supplemental Fig. S2*D*), likely, because of contribution of substrate stiffness or nonlinear behavior of nuclei due to chromatin or biopolymer networks. Thus, throughout the analyses, an indentation depth <1 μm was considered.

To verify whether 1 μm indentation was comprised within the recommended limits for the Hertz model application (*i.e.*, 10% of sample thickness), we reconstructed nuclear topography by force mapping (Supplemental Fig. S2*A*) (34, 39) and determined the maximum nuclear thickness. A collection of F-D curves was acquired on a matrix of 40 × 40 points that were equally distributed on a 30 × 30 μm^2^ square area. Silicon nitride triangular cantilevers with pyramidal tips, a 20 nm nominal tip radius and typical elastic constant of 0.06 N/m were used (NP; Bruker Corporation, Billerica, MA, USA). A maximal force of 0.20 nN was applied. The sample topography was reconstructed from the analysis of the cantilever deflection and corrected for the indentation depth that was calculated for each point of the map. The maximum height of fibroblast nuclei was calculated on the peak cross-section of the AFM topography. Isolated nuclei appear as spherical caps with a high curvature radius due to the interaction with the substrate. The maximum nuclear thickness of nuclei that were extracted from CTR fibroblasts was 12.35 ± 0.33 μm (*n*=19 nuclei) and was constant in the surrounding area (±8.85% variance at ±2 μm from the maximum). The maximum indentation depth of 1 μm represents 8% of the sample thickness and is within the values recommended for the Hertz model (37).

### Patch-clamp recordings

Nuclei were isolated by osmotic lysis as described above. The nuclear pellet was resuspended in 1 ml of external solution and kept on ice until plating. Patch-clamp recordings were performed on isolated nuclei using standard methods (40, 41). In brief, the patch electrodes were pulled from hard borosilicate glass (Heidelberg, Germany) on a Brown-Flaming P-87 puller (Sutter Instruments, Novato, CA, USA). The pipettes were fire polished to an external tip diameter of 1–1.5 μm and 7–10 MΩ electrodes resistance. We applied the standard patch-clamp technique to obtain nucleus-attached patches between 20 and 50 GΩ in resistance. The external solution was also used to fill the recording patch-clamp electrode and was composed by the following (in mM): 120 KCl, 2 MgCl_2_, 0.1 CaCl_2_, 1.1 EGTA (final Ca^2+^ concentration of 10^−4^), and 10 HEPES at pH 7.4. An Axopatch 200 B amplifier and PClamp 9 (both from Molecular Devices, Sunnyvale, CA, USA) were used to record and analyze the single channel currents. Current recordings were digitized at 5 KHz and filtered at 1000 Hz.

### Statistical analysis

The statistical analysis between the groups with normal distributions was performed using Student's *t* test for 2 groups or ANOVA for multiple comparisons. When the normality test failed, the analysis was performed using nonparametric tests, such as the Mann-Whitney rank sum test or Kruskal-Wallis 1-way ANOVA on ranks followed by a *post hoc* Dunn's test. The differences between groups were considered to be statistically significant when *P* < 0.05. Data throughout the text are reported as average values ± se, except when otherwise specified.

## RESULTS

### LB1 is overexpressed in ADLD fibroblasts

**Figure 1.**
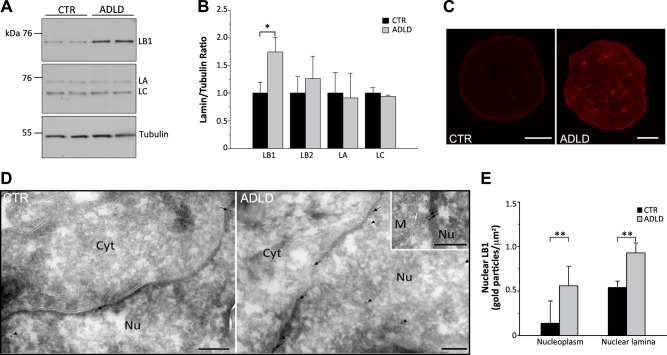
LB1 was overexpressed in ADLD primary human skin fibroblasts. LB1 protein expression was analyzed in primary human skin fibroblasts that were derived from patients with ADLD and CTR subjects. *A)* Representative Western blots of LB1 and LA/C in fibroblast total lysates. γ-Tubulin was used as the loading CTR. *B)* Quantitative analysis of LB1, LB2, and LA/C expression levels. The data are normalized to γ-tubulin and expressed as percentages of CTR levels. The bars represent the mean ± se. of 3–6 independent experiments. *P < 0.05, Student's *t* test. *C)* Representative maximal projections of z-stack confocal images of nuclei from CTR and ADLD human skin fibroblasts immunostained for LB1. Scale bar = 5 μm. *D)* Representative immunoelectron microscopy images from CTR (left panel) and ADLD (right panel) nuclei immunodecorated for LB1. Arrows and arrowheads indicate gold particles at the nuclear lamina and in the nucleoplasm, respectively. In the inset, the 2 arrows indicate a cluster of immunogold particles at the nuclear envelope of one ADLD nucleus. Cyt = cytosol; M = mitochondrion; Nu = nucleus. Scale bars = 200 nm. *E)* Semiquantitative analysis of the density of gold particles detected at the nuclear lamina and nucleoplasm of CTR and ADLD nuclei. Values represent the mean ± se. **P < 0.01, Student's *t* test.

We first analyzed LB1 expression in skin fibroblasts from ADLD patients who carry a duplication of the LB1 gene, siblings without this duplication, and age-matched healthy volunteers. LB1 expression was similar in fibroblasts from healthy volunteers and noncarrier siblings (LB1/tubulin ratio, average in healthy volunteers: 1.00±0.20; noncarrier siblings: 0.96±0.22; *P*=0.46, Student's *t* test), which were therefore pooled as CTRs. Compared with these CTRs, fibroblasts from ADLD patients exhibited normal levels of the cognate proteins LB2, LA, and LC but significantly overexpressed LB1 (**Fig. 1*A***–***C***).

We next investigated whether the subcellular localization of LB1 is altered in ADLD cells. We found that LB1 is correctly localized to the nuclear lamina and the nucleoplasm in both ADLD and CTR nuclei (Fig. 1*C*). However, immunofluorescence and immunoelectron microscopy revealed that in ADLD nuclei, LB1 immunoreactivity is significantly increased in both the nuclear lamina and the nucleoplasm (Fig. 1*C-E*). Clusters of gold particles were frequently observed underneath the nuclear envelope of ADLD, but not CTR, nuclei, indicating that the overexpressed protein had accumulated in *foci* (Fig. 1*D*, inset). Consistent with the EM data (Fig. 1*D*, *E*), the sequential biochemical extraction of nuclear proteins with increasing salt concentration revealed that the majority of LB1 was in the insoluble fraction of both CTR and ADLD fibroblasts, indicating that LB1 primarily associates with the nuclear lamina (Supplemental Fig. S3*A*). In ADLD fibroblasts, the level of LB1 in the insoluble fraction was significantly higher than that in CTR cells (Supplemental Fig. S3*A*). Overall, these experiments indicate that overexpressed LB1 mainly localizes to the nuclear lamina, where it accumulates in *foci*.

### LB1 overexpression does not affect cell proliferation in ADLD fibroblasts

Cell proliferation can affect nuclear mechanics (33), and LB1 expression levels regulate the cell proliferation rate in lung embryonic fibroblasts (23, 42) and human dermal fibroblasts (43). We therefore first examined whether LB1 overexpression was associated with altered proliferation in ADLD fibroblasts. The PD over 20 passages *in vitro* was similar in primary fibroblasts from CTR subjects (*P*=0.706, 1-way ANOVA, *n*=6) and those from ADLD patients (*P*=0.801, 1-way ANOVA, *n*=2; Supplemental Fig. S3*C*). Consistently, the average PD and doubling time (DT) of CTR and ADLD fibroblasts were comparable. The average PD in CTR fibroblasts was 1.38 ± 0.57; in ADLD fibroblasts, 1.47 ± 0.51 (*P*=0.494, Student's *t* test). The average DT of CTR fibroblasts was 6.34 ± 3.71 d; ADLD fibroblasts, 5.84 ± 3.56 d (*P*=0.544, Mann-Whitney rank sum test). Overall therefore, ADLD fibroblasts had no obvious proliferation abnormalities.

To investigate how ADLD fibroblasts adapt their proliferative potential to changes in environmental growth factors, we assessed their ability to enter quiescence on serum withdrawal for 24 h. Serum deprivation for 24 h caused a significant reduction in BrdU incorporation in both CTR and ADLD fibroblasts, indicating a quiescent phenotype. The percentage of BrdU-positive cells in proliferating and quiescent conditions was comparable in CTR and ADLD fibroblast cultures (Supplemental Fig. S3*D*), confirming their equal proliferative features. Notably, the quiescent state could be reversed by reexposing the cells to serum-containing medium for 24 h, even after prolonged (5 d) serum withdrawal. Indeed, after 5 day serum deprivation, 4.80 ± 1.30% of CTR cells and 1.57 ± 0.02% of ADLD cells were BrdU positive, consistent with a quiescent state. Reexposing such quiescent cells to serum for 24 h increased the proportion of BrdU-positive cells to 34.96 ± 5.01% in CTR cells and 33.33 ± 8.2% in ADLD cells, indicating the absence of senescence in both CTR and ADLD cultures. Overall, these results indicate that ADLD fibroblasts normally adapt to the absence of growth stimuli by entering a quiescent state, without undergoing senescence.

### LB1 overexpression increases nuclear rigidity in ADLD human skin fibroblasts

**Figure 2.**
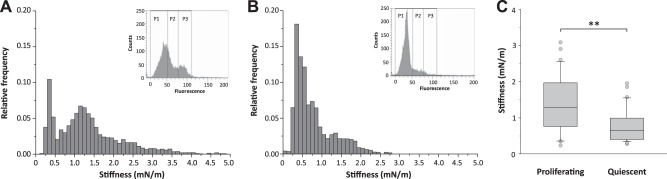
Nuclear stiffness of human skin fibroblasts varied with the cell proliferation state. AFM nuclear stiffness analysis was performed on nuclei that were extracted from proliferating (*A*) and quiescent CTR human skin fibroblasts (*B*). *A*, *B)* Stiffness distribution calculated from F-I curves; *y-*axis: relative frequency values (0<f<1); *x*-axis: stiffness bins (0.1 mN/m). The insets in *A* and *B* show the FACS analysis of propidium iodide-labeled fibroblasts, indicating that the majority of cells are in G_1_/S phases (P1) in quiescent cells (*B*). *C)* Box plot of average nuclear stiffness values. A total of 65 nuclei of proliferating (*n*=31) and quiescent (*n*=34) fibroblasts were analyzed in 2 independent experimental sessions. **P < 0.01, Mann-Whitney rank sum test.

We next aimed to determine whether LB1 overexpression in ADLD cells affects the elastic properties of the nucleus. A cell's mechanical properties (33, 44) and LB1 levels (45) vary during the cell cycle. It is therefore also possible that the mechanical properties of isolated nuclei differ according to a cell's proliferative state. To test this and thus define the optimal proliferative state for nuclear stiffness analysis, we performed AFM indentation on intact nuclei isolated from CTR proliferating or quiescent fibroblasts. Nuclei from proliferating cells displayed a broader probability distribution of stiffness values [95% confidence Interval, CI: 0.25÷3.55 mN/m; **Fig. 2*A***] than nuclei from quiescent fibroblasts (95% CI: 0.25÷2.05 mN/m; Fig. 2*B*). Moreover, nuclei from proliferating cells displayed a bimodal distribution, indicating that there is a subpopulation of cells with lower elastic modulus, likely the cells in G_1_/S (P1 population of flow cytometry data; Fig. 2*A*, inset). Further, the average stiffness of proliferating nuclei was significantly higher than that of quiescent nuclei (Fig. 2*C*). These results indicate that the nuclear mechanics change with a cell's proliferative state.

We also tested whether nuclear stiffness varies with aging in culture by analyzing quiescent CTR fibroblasts at different passages. We observed no significant differences in the calculated stiffness values at passages 4–16. The mean stiffness at passage 4 was 0.71 ± 0.08 mN/m (*n*=4); at passage 8, 0.76 ± 0.10 mN/m (*n*=21); and at passage 16, 0.57 ± 0.16 mN/m (*n*=7; *P*=0.311, Kruskal-Wallis 1-way ANOVA on ranks), indicating that aging *in vitro* ≤16 passages does not affect nuclear stiffness. Based on these results, to reduce the intrinsic variability and exclude confounding effects arising from cell cycle stages, ADLD and CTR nuclear rigidity was analyzed in nuclei isolated from quiescent fibroblasts within 16 passages.

**Figure 3.**
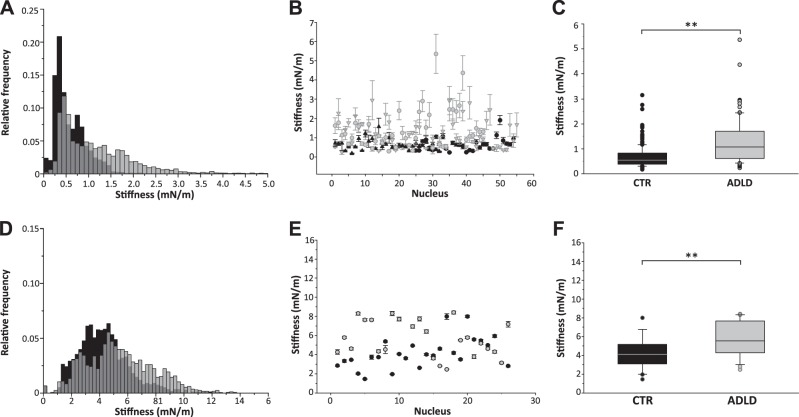
Nuclear stiffness was increased in ADLD human skin fibroblasts. AFM nuclear stiffness analysis was performed on extracted fibroblast nuclei (*A-C*) or living quiescent CTR and ADLD fibroblasts (*C*, *D*). *A)* Representative stiffness distributions that were calculated from the F-I curves of nuclei from fibroblasts of one CTR (black) and one ADLD (gray) patient; *y*-axis: relative frequency values (0<f<1); *x-*axis, stiffness bins (0.1 mN/m). *B)* Average calculated stiffness of nuclei from fibroblasts of 2 CTR subjects (black symbols) and 2 patients with ADLD (gray symbols). Each symbol represents the average stiffness ± sd of 1 nucleus. *C)* Box plot of average nuclear stiffness values of CTR and ADLD fibroblasts. A total of 291 fibroblast nuclei from 6 CTR subjects (*n*=191 nuclei) and 2 ADLD (*n*=100 nuclei) patients were analyzed in 3–6 independent experimental sessions. **P < 0.01, Mann-Whitney rank sum test. *D)* Representative stiffness distributions that were calculated from the F-I curves of living fibroblasts of 1 CTR (black) and 1 ADLD (gray) patient; *y*-axis: relative frequency values (0<f<1); *x*-axis: stiffness bins (0.1 mN/m). *E)* Average calculated stiffness of nuclei from living fibroblasts of CTR (black symbols) and ADLD subjects (gray symbols). Each symbol represents the average stiffness ± sd of 1 cell nucleus. *F)* Box plot of average nuclear stiffness values of living CTR and ADLD fibroblasts. A total of 51 nuclei from living CTR (*n*=25) and ADLD (*n*=26) fibroblasts were probed in 4 independent experimental sessions. **P < 0.01, Mann-Whitney rank sum test.

The nuclear stiffness values in ADLD nuclei had a broader frequency distribution than those of CTR nuclei (95% CI, CTR 0.15÷1.45 mN/m; ADLD: 0.25÷3.45 mN/m) (**Fig. 3*A***). The majority of ADLD nuclei (77%) showed stiffness values higher than the median CTR stiffness value (Fig. 3*B*). Moreover, the median nuclear stiffness was significantly higher in ADLD nuclei than CTR nuclei (Fig. 3*C*). Consistent with this, the Young's elastic modulus (E) of ADLD nuclei was 2.7-fold higher than CTR nuclei (CTR, 236.68±16.33 Pa, *n*=191; ADLD, 644.06±65.07 Pa, *n*=100; *P* < 0.01, Student's *t* test). To explore whether nuclear stiffness displays a similar behavior in living cells, we performed AFM indentation on adherent living CTR and ADLD fibroblasts in quiescent conditions. The nuclear stiffness of living fibroblasts was ∼6-fold higher than that of isolated nuclei, suggesting significant contributions from adhesive forces, the underlying cytoskeleton, and cytosolic factors to the measurement. Consistent with results on isolated nuclei, we found that the nuclear stiffness was significantly increased in living ADLD fibroblasts (Fig. 3*D-F*). In fact, ADLD living fibroblasts had a broader frequency distribution of nuclear stiffness values than CTR nuclei (Fig. 3*D*). The majority of ADLD fibroblasts (81%) showed nuclear stiffness values higher than the median CTR stiffness value (Fig. 3*E*) and the average nuclear stiffness was significantly increased (+73%) in ADLD nuclei (Fig. 3*F*). Overall, these results indicate that in somatic cells from ADLD patients, pathological LB1 overexpression is linked to increased nuclear rigidity.

### ADLD-associated increased nuclear rigidity is dependent on LB1 overexpression

**Figure 4.**
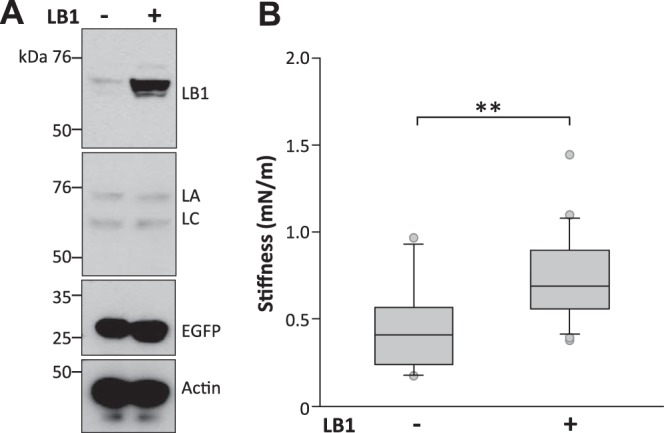
Transient LB1 overexpression increased nuclear stiffness in HEK293 cells. AFM nuclear stiffness analysis of nuclei isolated from HEK293 cells expressing both LB1 and EGFP or EGFP alone. *A)* Representative Western blots of LB1, LA/C, EGFP, and actin in total lysates from cells transiently overexpressing both LB1 and EGFP or EGFP alone. *B)* Box plot of average nuclear stiffness of HEK293 cells overexpressing both LB1 and EGFP or EGFP alone. A total of 39 nuclei from cells expressing LB1/EGFP (*n*=23) or EGFP alone (*n*=16) were analyzed in 4 independent experimental sessions. **P < 0.01, Mann-Whitney rank sum test.

**Figure 5.**
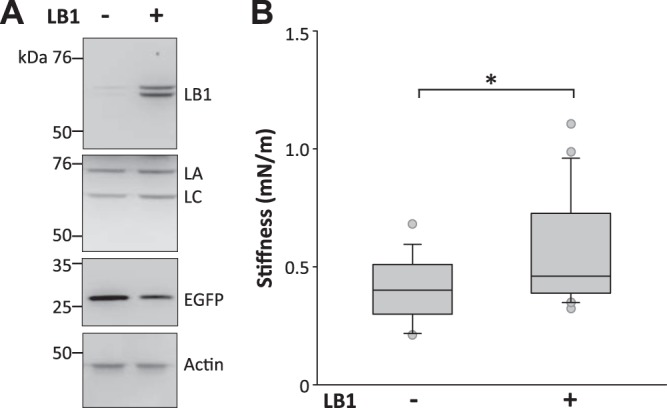
Transient LB1 overexpression increased nuclear stiffness in living N2a neuronal cells. AFM nuclear stiffness analysis in living N2a neuronal cells expressing both LB1 and EGFP or EGFP alone. *A)* Representative Western blots of LB1, LA/C, EGFP, and actin in total lysates from cells transiently overexpressing both LB1 and EGFP or EGFP alone. *B)* Box plot of average nuclear stiffness of N2a cells overexpressing both LB1 and EGFP or EGFP alone. A total of 38 living N2a cells expressing LB1/EGFP (*n*=21) or EGFP alone (*n*=17) was analyzed in 4 independent experimental sessions. *P < 0.05, Mann-Whitney rank sum test.

To test whether LB1 regulates nuclear mechanics, we next examined whether transient LB1 overexpression mimics the pathological phenotype of ADLD and increases nuclear rigidity. Using lipofection, we transfected HEK293 cells with bicistronic expression vectors containing cDNA for LB1 plus an EGFP reporter or the EGFP reporter alone (**Fig. 4*A***). EGFP-positive cells were isolated 48 h later by FACS, and the nuclei were extracted for AFM analysis. Nuclear stiffness was significantly higher in nuclei overexpressing both LB1 and EGFP compared with nuclei expressing EGFP alone, displaying a 70% increase in the median stiffness (Fig. 4*B*). To confirm this result in disease target cells, we tested if LB1 overexpression in neuronal cells caused a similar effect. In N2a neuronal cells, transient LB1 overexpression increased nuclear stiffness of isolated, FACS-sorted nuclei (mean stiffness of LB1/EGFP, 2.28±0.60 mN/m; EGFP alone, 1.19±0.30 mN/m; *P*<0.01, Mann-Whitney rank sum test) and in living adherent cells (**Fig. 5*B***). The levels of the cognate proteins LA/C were not changed by LB1 overexpression in both HEK293 and N2a cells (Figs. 4*A, 5A*). These results support the hypothesis that LB1 is directly involved in increasing the nuclear rigidity associated with ADLD.

**Figure 6.**
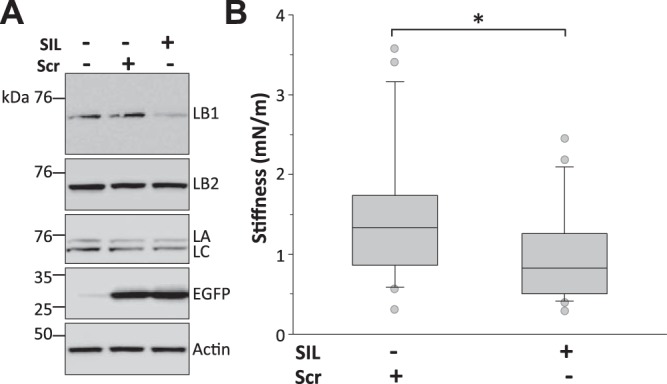
LB1 knockdown reduced the nuclear stiffness of ADLD fibroblasts. AFM nuclear stiffness analysis of ADLD fibroblast nuclei following LB1 silencing with LB1 shRNA/EGFP plasmid (SIL) or transfection with scrambled shRNA/EGFP (Scr). *A)* Representative Western blots of LB1, LB2, LA/C, EGFP, and actin in total lysates of HEK293 cells 48 h following transfection of SIL or Scr plasmids. LB1 shRNA reduced LB1 expression without altering the levels of cognate lamins. *B)* Box plot of average nuclear stiffness of ADLD fibroblasts on LB1 silencing. A total of 51 nuclei from EGFP expressing cells (SIL, *n*=27; Scr, *n*=24) were analyzed in 2 independent experimental sessions. *P < 0.05, Mann-Whitney rank sum test.

To investigate whether the increased nuclear stiffness in ADLD fibroblasts was specifically due to LB1 overexpression, we knocked down LB1 in these cells and examined nuclear rigidity. To silence LB1 we transfected a mixture of 2 plasmids bearing different LB1 shRNA sequences (23, 24) or a scrambled RNA plasmid by amaxa nucleofection. Both the shRNA and scrambled plasmids also contained the cDNA for the EGFP reporter. To confirm the knockdown, lamin protein levels were determined in EGFP-positive cells selected by FACS 72 h after transfection (**Fig. 6*A***). LB1 levels were reduced by 54% in cells transfected with LB1 shRNA compared with samples transfected with scrambled shRNA: the LB1/actin ratio after transfection with the scrambled plasmid was 0.88 ± 0.02 and was reduced by LB1 shRNA to 0.39 ± 0.06 (*P*<0.01, Student's *t* test). The levels of cognate lamins were unaltered (LB2/actin ratio for scrambled, 0.80±0.04, for LB1 shRNA, 0.79±0.01; LA/actin ratio for scrambled, 0.44±0.08, for LB1 shRNA, 0.36±0.09; LC/actin ratio for scrambled, 0.52±0.01, for LB1 shRNA, 0.40±0.05; n.s., Student's *t* test).

We then measured the stiffness of nuclei with AFM indentation. Nuclei from ADLD fibroblasts transfected with scrambled RNA had the same stiffness as untransfected cells (mean stiffness of ADLD plus scrambled, 1.42±0.16 mN/m, *n*=27; ADLD untransfected, 1.18±0.10 mN/m, *n*=55; *P*=0.14 Mann-Whitney rank sum test). However, LB1 silencing significantly reduced the rigidity of ADLD nuclei (Fig. 6*B*), restoring them to levels indistinguishable from CTR nuclei (ADLD silenced, 1.01±0.13 mN/m, *n*=24; CTR untransfected, 0.83±0.08 mN/m, *n*=42; *P*=0.19, Mann-Whitney rank sum test). This indicates that the increased stiffness of ADLD nuclei is specifically linked to LB1 overexpression.

Consistent with previous findings (14), the nuclear rigidity of LB1-null MEFs (LB1Δ/Δ) (20) was similar to wild-type MEFs (Supplemental Fig. S4*B*), indicating that removal of LB1 alone does not reduce nuclear stiffness below CTR values.

### LB1 overexpression does not affect nuclear protein localization in ADLD fibroblasts

**Figure 7.**
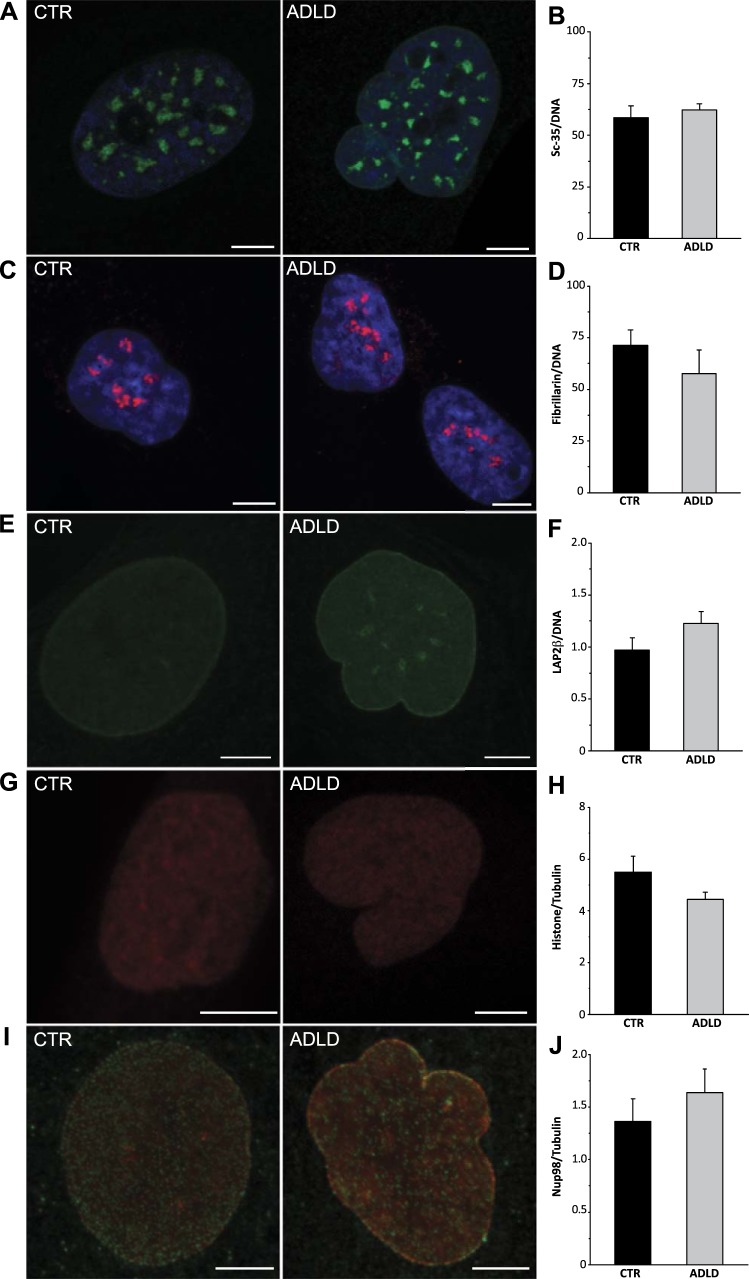
Localization and expression of selected nuclear proteins were unaltered in ADLD fibroblasts. *A*, *C*, *E*, *G*, *I)* Representative maximal projections of z-stack confocal images of nuclei from CTR and ADLD human skin fibroblasts immunoreactive for sc-35 (green; *A*), fibrillarin (red; *C*), LAP2β (*E*), trimethyl-histone-H3(Lys 27) (*G*), nuclear pore complex (*I*), and LB1 (red, I). In *A* and *C*, nuclear DNA is counterstained with Hoechst-33342 (blue). Scale bars = 5 μm. *B*, *D*, *F)* Quantification of sc-35 (*B*), fibrillarin (*D*), and LAP2β (μ) immunoreactive area normalized on area of DNA staining with Hoechst-33342. *H*, *J)* Quantification of protein expression levels of trimethyl-histone-H3(Lys 27) (*H*) and nucleoporin Nup98 (*J*) by Western blot analysis. Values are normalized on β-tubulin expression.

To evaluate whether LB1 overexpression affects nuclear architecture and increases nuclear stiffness by regulating protein localization, we analyzed the expression and localization of key nuclear proteins in CTR and ADLD fibroblasts by immunohistochemistry. These included nuclear proteins, such as the splicing factor sc-35, the nucleolar protein fibrillarin, chromatin-associated trimethyl histone H3, nuclear pore complexes, and the LB1-interacting proteins Nup153 and LAP2β. In our experimental conditions, although there was an increased proportion of misshapen nuclei (Supplemental Fig. S3*B*) in ADLD fibroblasts and increased LB1 levels (LB1 immunoreactive area/DNA stained area ratio, CTR 84.9±5.9; ADLD 185.0±10.8; *P*<0.01), we did not observe any gross abnormalities of the localization and levels of sc-35 (**Fig. 7*A***, ***B***), fibrillarin (Fig. 7*C*, *D*), LAP2β (Fig. 7*E*-F), trimethyl histone H3 (Fig. 7*G*, *H*), and nucleoporins (Fig. 7*I*, *J*). Furthermore, we did not detect any significant differences in the proportion of nuclei with activated RNA polymerase II (percentage of nuclei immunoreactive for RNA polymerase II in CTR, 26.17±2.60%, *n*=767; in ADLD, 28.33±12.42%, *n*=317; n.s., Student's *t* test), consistent with overall integrity of the LB1-dependent nucleoskeleton (46). These data support the view that increased nuclear stiffness in ADLD fibroblasts is not due to global changes in nuclear protein localization.

### LB1 overexpression in ADLD fibroblasts is associated with altered nuclear ionic permeability

**Figure 8.**
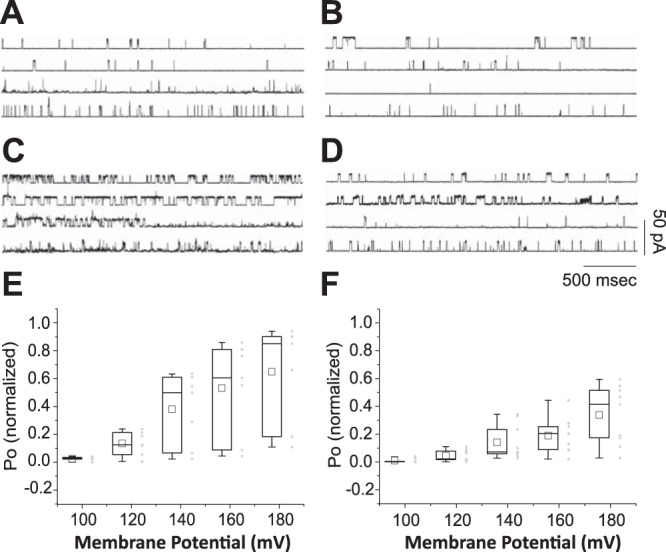
Nuclear ionic permeability was altered in ADLD fibroblasts. Single-channel recordings in isolated nuclei. *A–D)* Selected traces of nucleus-attached single-channel activity in isolated nuclei from CTR (*A*, *C*) and ADLD (*B*, *D*) fibroblasts challenged with 100 mV (*A*, *B*) and 140 mV (*C*, *D*) test potentials. *E*, *F)* Channel open probability. The channel open probability in CTR nuclei (*E*) increased from 0.02 ± 0.005 at 100 mV to 0.64 ± 0.14 at 180 mV (*n*=7). In ADLD nuclei (*F*), the channel open probability displayed a normalized value of 0.009 ± 0.004 at 100 mV and 0.34 ± 0.07 at 180 mV (*n*=9).

To evaluate whether the stiffness of ADLD nuclear lamina has functional consequences on the ionic permeability of nuclei, we performed on-nucleus patch-clamp experiments on isolated nuclei (41, 47). At low-voltage stimuli, single channels were not evident in either CTR or ADLD nuclei. To induce channel openings independently of molecular cytosolic factors and substrates, we challenged isolated nuclei with increasing voltage steps, from 100 to 180 mV. Under these conditions, we recorded channel opening in virtually all trials. Single channel conductance was similar in CTR and ADLD nuclei (average conductance of CTR, 75±3.4 pS; ADLD, 77±4.1 pS). However, the mean open time was significantly different in CTR and ADLD channels (**Fig. 8**). At all voltages applied, the mean open probability (Po) in ADLD nuclei was lower than in CTR nuclei [2-way ANOVA, genotype (*F*=23.785; *P*<0.001); treatment (*F*=19.541; *P*<0.001); genotype×treatment (*F*=2.713; *P*<0.05]), with statistically significant differences at membrane potentials equal or higher than 140 mV. The mean Po at 140 mV in CTR nuclei was 0.38 ± 0.10, in ADLD, 0.15 ± 0.04 (*P*<0.05); at 160 mV in CTR, 0.55 ± 0.13, in ADLD, 0.19 ± 0.04 (*P*<0.05); and at 180 mV in CTR, 0.64 ± 0.14, in ADLD 0.34 ± 0.07 (*P*<0.05). Overall, these results suggest that nuclear signaling through ion channels is inversely proportional to nuclear rigidity in ADLD fibroblasts.

## DISCUSSION

Here, we demonstrate that in primary skin fibroblasts derived from patients with ADLD, LB1 is overexpressed. In these cells, the LB1 protein primarily localizes to the nuclear lamina and causes increased nuclear stiffness. Transient overexpression of LB1 also increases nuclear stiffness in HEK293 and N2a neuronal cells, mimicking the ADLD nuclear phenotype. Notably, the expression of cognate lamins is unaltered in ADLD fibroblasts or LB1-transfected cells, indicating that the increased stiffness does not depend on these other lamins. Moreover, shRNA silencing in ADLD fibroblasts, which specifically reduces LB1 protein levels, restores nuclear elasticity to levels that are indistinguishable from CTR cells, supporting the view that LB1 overexpression is directly linked to enhanced nuclear rigidity. The increased stiffness of ADLD nuclei is associated with a reduced open probability of nuclear ion channels when voltage steps are applied, suggesting that LB1-driven changes in nuclear mechanics may functionally affect nuclear ionic signaling.

Our findings support the idea that lamin mutations or altered lamin expression significantly affect the elastic properties of the nuclear lamina. A- and B-type lamins form distinct, but interacting, stable networks in the lamina (24) and play distinct roles in defining the nuclear architecture and mechanics (14). Indeed, under biaxial strain, LA/C-deficient MEFs exhibit increased numbers of misshapen nuclei, increased nuclear deformation, and decreased cell viability (14). In contrast, while it does alter nuclear morphology, LB1 deficiency does not affect nuclear deformability in MEFs (14). Consistently, we found that the nuclear stiffness of LB1-null MEFs is not significantly different from that of wild-type MEFs. It is possible that the impact of LB1 deficiency could be masked by redundant roles of LB2, which is normally expressed in LB1-null MEFs (48). Alternatively, based on previous findings (14), we can postulate that, in cells expressing both A- and B-type lamins, LA is the principal determinant of nuclear deformability. Nonetheless, at constant levels of cognate lamins, LB1 overexpression increases the number of misshapen nuclei (Supplemetnal Fig. S3*B*) and significantly increases nuclear stiffness, indicating that LB1 protein over-dosage plays a key role in defining the biophysical properties of the nuclear lamina.

In agreement with previous evidence that cell mechanical properties vary during the cell cycle (33), we found that nuclear stiffness varies according to the proliferation state of cells: it is significantly higher in proliferating than quiescent fibroblasts. However, the LB1-induced increased stiffness is independent of cell proliferative state: AFM indentation measurements on nuclei from human fibroblasts in a quiescent state rule out a confounding effect of cell proliferation. Moreover, although there is substantial variation among fibroblasts obtained from different donors, the average proliferative rates and the ability to enter quiescence with serum withdrawal are similar in ADLD and CTR fibroblasts. This contrasts with previous evidence that altered LB1 protein levels may affect cell proliferation. In fact, it has been shown that LB1 overexpression both decreases (43) and increases (23) the proliferation of human fibroblasts of dermal or embryonic lung origin, respectively. Several factors may contribute to these divergent results, including developmental stage (embryonic *vs.* adult), tissue of origin (lung *vs.* skin), aging *in vitro*, transient overexpression of the protein, and/or interactions with the genetic background of distinct donors. In our study, we compared primary human skin fibroblasts derived from different adult donor patients or age-matched CTR subjects, while studies from other groups (23, 43) experimentally manipulated LB1 levels in cells with uniform genetic backgrounds (*e.g.*, WI-38 cells). In view of the complex regulation of cell proliferation, it is possible that individual genetic variables may have masked the specific effects of LB1 in our study.

The mechanisms by which excess LB1 causes ADLD pathology are unclear. One hypothesis is that LB1 overexpression could affect nuclear protein organization, increasing the stiffness of nuclei as a consequence of chromatin and protein relocalization. However, our results do not support this idea. Although the proportion of misshapen nuclei in ADLD fibroblasts is increased, we did not observe any gross abnormalities of nuclear architecture based on the localization of nuclear proteins, including sc-35, fibrillarin, histone H3, LAP2β and nucleoporins, or chromatin, as detected by Hoechst 33342 DNA staining and electron microscopy. Our findings differ from those of a previous study, which reported that the transfection of LB1 in cell lines of neurons, astrocytes, and oligodendrocytes leads to severe abnormalities of nuclear morphology and altered LAP2 levels and localization (49). The use of primary human skin fibroblast cultures *vs.* transiently transfected cell lines may account for this discrepancy. Indeed, the protein expression achieved by transient transfection in cell lines may be severalfold higher than physiological levels. In contrast, LB1 overexpression in primary human fibroblasts of patients with ADLD is mild (*i.e.*, 1.5 to 2-fold increase) and may translate into only subtle changes in the localization or activation of signaling molecules.

An alternate model is that the LB1-induced increase in nuclear rigidity may alter cells' nuclear signaling (for a review, see refs. 50, 51). The nucleus, because of its mechanical properties and interconnections with the cytoskeleton, can act as a mechanosensor of internal and external forces, converting these signals into biological responses by activating gene expression (52, 53). Consistent with this, disrupting the nuclear lamina results in nuclear abnormalities and altered mechanotransduction signaling. Indeed, in LA-deficient MEFs (48), the mechanical activation of NF-κB is compromised, and cells lacking emerin or LA have reduced expression of mechanosensitive genes (48, 54). Our results support this link between ADLD and improper nuclear signal transduction and further suggest that signaling defects depend on nuclear ion channel function. We recently reported that in ADLD fibroblasts Oct1 recruitment to the nuclear periphery is increased and its mobilization in response to oxidative stress is reduced (55). In nuclei isolated from ADLD cells, channel openings induced by biophysical challenge (*i.e.*, voltage steps on isolated nuclei, in the absence of molecular cytosolic factors and substrates) are reduced, supporting the view that altered mechanical properties of the nuclear lamina may translate into functional changes in nuclear signaling. This is consistent with findings demonstrating that, in osteoblasts (56) and HEK293 cells (57), membrane strain and tension affect the open probability of mechanosensitive ion channels. While these results implicate nuclear signaling in ADLD, further experiments are needed to identify the specific mechanisms of altered nuclear ionic permeability that are affected by LB1-induced nuclear rigidity.

Notably, LB1 protein levels also modulate nuclear rigidity in neuronal N2a cells. This is specifically relevant for the pathogenesis of ADLD, which affects the central nervous system. There is some evidence that mechanical forces and neuronal function are linked (58). For example, in primary hippocampal neurons, nuclear geometry is dynamically regulated by neuronal activity and death signals, which modulates nuclear infolding and nuclear calcium transients in opposite directions (59). Nuclear calcium is the signal that switches on the gene expression required for the long-term implementation of brain functions, such as learning and memory, and adaptive responses of the nervous system, such as activity-dependent neuron survival and neural repair after injury (60). Given this scenario and the evidence that ADLD fibroblasts have an increased number of misshapen nuclei, we postulate that changes in nuclear stiffness in neurons may alter their nuclear geometry, thus affecting neuronal survival and function in ADLD.

In conclusion, our findings reveal a previously unrecognized function of LB1 in controlling the mechanical properties of cell nuclei. LB1 overexpression alters nuclear stiffness in human somatic cells, possibly leading to changes in ionic signaling in ADLD cells. In ADLD fibroblasts, LB1 knockdown restores nuclear elasticity to physiological levels, suggesting that regulating LB1 expression may represent a target for the discovery of therapies for ADLD. Further investigation is required to elucidate precisely how LB1 overexpression affects neuronal nuclear signaling and drives cerebral pathogenic cascades in ADLD.

## Supplementary Material

Supplemental Data
