# Supplementary material for: Lamin B1 overexpression increases nuclear rigidity in autosomal dominant leukodystrophy fibroblasts
Source: FASEB J. 2014 Sep;28(9):3906–18. doi: 10.1096/fj.13-247635 (PMC4139899; doi:10.1096/fj.13-247635)
Supplement: Supplemental Data [file supp_28_9_3906__index.html]

Lamin B1 overexpression increases nuclear rigidity in autosomal dominant leukodystrophy fibroblasts — Supplemental Data 

# Lamin B1 overexpression increases nuclear rigidity in autosomal dominant leukodystrophy fibroblasts

## Supplemental Data

**Files in this Data Supplement:**

- Supplemental Data - (*13-247635SuppData.zip; compressed file 2.24 MB*)

- Facebook
- Google+
- LinkedIn
- Mendeley
- Reddit
- StumbleUpon
- Twitter

What's this?
