# Supplementary material for: Lamin B1 overexpression increases nuclear rigidity in autosomal dominant leukodystrophy fibroblasts
Source: FASEB J. 2014 Sep;28(9):3906–18. doi: 10.1096/fj.13-247635 (PMC4139899; doi:10.1096/fj.13-247635)
Supplement: Supplemental Data [file supp_fj.13-247635_13-247635SuppData.zip › Suppl.Fig. S1.pdf]

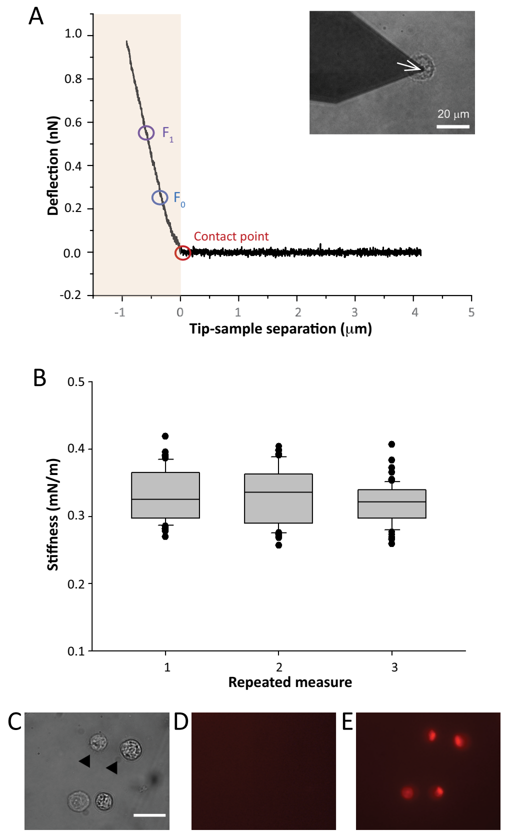

Figure S1 - AFM force spectroscopy of isolated nuclei did not disrupt nuclear integrity.
(A) Typical force curve obtained by AFM force spectroscopy on isolated nuclei. The negative values in the X-axis (pink-shaded area) highlight the indentation of the spherical probe within the nucleus. The slope of the linear fit between 0.25 nN (F_0) and 0.55 nN (F_1) force values represents the stiffness. *Inset*: optical image of a nucleus beneath the AFM probe. The spherical indenter is displayed at the cantilever free end (white arrowhead). (B) Acquisition of multiple maps on the same nucleus did not damage the nucleus. Three consecutive 8 x 8 point maps (64-force curves) were acquired at the same position on one nucleus. Each box represents the average stiffness ± SD of one map. The values were not significantly different ($p = 0.20$, Kruskal–Wallis One-way Analysis of Variance on Ranks), indicating the reproducibility of the measure and the integrity of the nucleus after each measurement. (C-E) Nuclear integrity was evaluated by exposure of naïve nuclei or nuclei that underwent AFM analysis (arrowheads) to 50 μg/ml propidium iodide (PI). Negative PI staining indicates that nuclei are intact following AFM analysis (D). Both naïve and AFM-measured nuclei were stained by PI following permeabilization with 1% Triton X-100 (E). Representative phase contrast (C) and fluorescence images (D-E) are shown. Scale bar: 20 μm.
