# Supplementary material for: Lamin B1 overexpression increases nuclear rigidity in autosomal dominant leukodystrophy fibroblasts
Source: FASEB J. 2014 Sep;28(9):3906–18. doi: 10.1096/fj.13-247635 (PMC4139899; doi:10.1096/fj.13-247635)
Supplement: Supplemental Data [file supp_fj.13-247635_13-247635SuppData.zip › Suppl.Fig. S2.pdf]

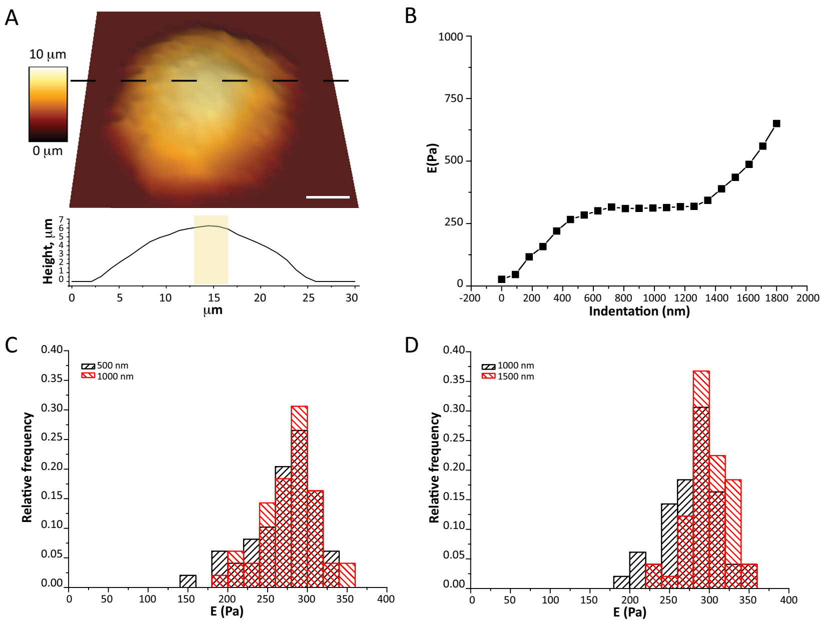

Figure S2 - Young's elastic modulus (E) as a function of sample indentation.
(A) Topographical reconstruction of one isolated nucleus derived from 40 x 40 F–D curves (n = 1600) acquired on a 30 x 30 μm^2 square area. To evaluate the maximum height for each nucleus, the peak cross-section (*i.e.,* black dotted line) was generated on each nucleus along the fast scan direction. The cross-section profile of the represented nucleus is shown in the graph. The yellow-shaded region in the graph corresponds to the area selected for the stiffness analysis. The thickness of the center (maximum height, average ± SEM: 12.35 ± 0.33 μm) was comparable with the thickness at the edges (11.25 ± 0.37 μm; n.s., Mann–Whitney Rank Sum Test, n = 19 nuclei) of the measured region, with an average variation of ± 8.85 %. (B-D) Variation of Young's elastic modulus (E) at different indentation depths. (B) The typical variation of E with increasing indentation is shown. When the indenter contacts the nuclear surface, E rose sharply to plateau values, which represent E for the nucleus. Further increases in indentation as determined by an additional increase in E were due to mixed contributions from both the nucleus and the substrate. (C-D) Relative frequency distribution of E for different indentation depths. The frequency distributions of E are comparable at indentations ≤1 μm (C) and shifted to higher values when the indentation depth increased to 1.5 μm (D).
