# Supplementary material for: Lamin B1 overexpression increases nuclear rigidity in autosomal dominant leukodystrophy fibroblasts
Source: FASEB J. 2014 Sep;28(9):3906–18. doi: 10.1096/fj.13-247635 (PMC4139899; doi:10.1096/fj.13-247635)
Supplement: Supplemental Data [file supp_fj.13-247635_13-247635SuppData.zip › Suppl.Fig. S3.pdf]

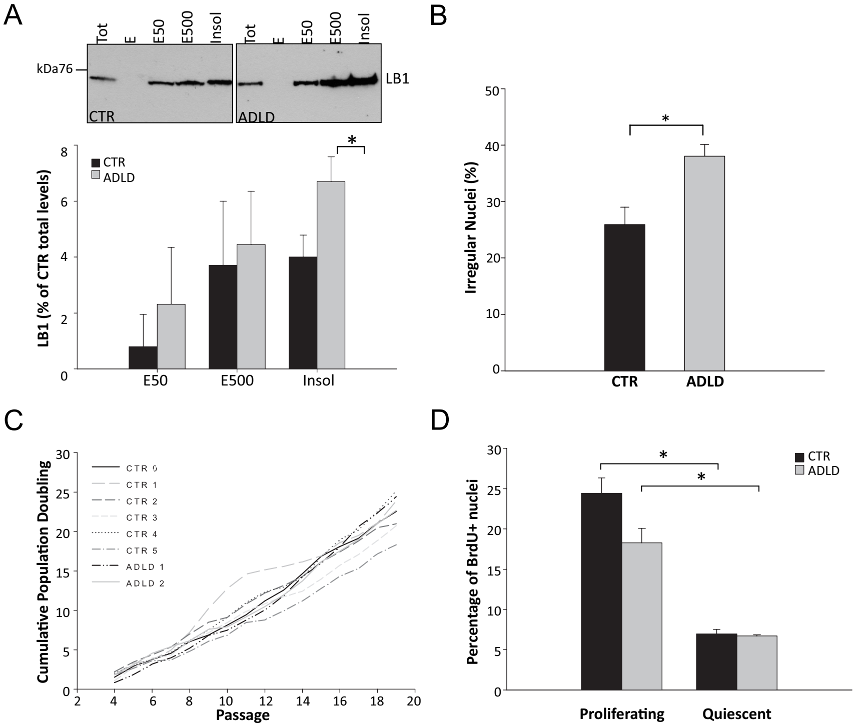

Figure S3 - ADLD fibroblasts displayed increased levels of nuclear insoluble LB1, higher number of misshapen nuclei than CTR, but normal proliferation.

(A) Western blot and quantitative analyses of LB1 levels in salt-extracted fractions of CTR and ADLD nuclei. *$p < 0.05$ ANOVA followed by Student–Newman–Keuls test. (B). Quantitative analyses of nuclear morphology. The percentage of misshapen nuclei was increased in ADLD fibroblasts compared to CTR fibroblasts. *$p < 0.05$, Student's t-test. (C) Cumulative population doubling curves of CTR and ADLD fibroblasts that were cultured *in vitro* for 20 passages. (D) BrdU incorporation in proliferating and quiescent CTR and ADLD human skin fibroblasts. A total number of 46,026 nuclei from primary fibroblasts of 6 CTR subjects (n = 33,134) and 2 patients with ADLD (n = 12,892) were analyzed in 6 independent experimental sessions. The data represent the percentage of BrdU-positive ($BrdU^+$) nuclei and are expressed as the mean ± SEM **$p < 0.05$ two-way ANOVA followed by Student–Newman–Keuls pairwise test.
