# Supplementary material for: Lamin B1 overexpression increases nuclear rigidity in autosomal dominant leukodystrophy fibroblasts
Source: FASEB J. 2014 Sep;28(9):3906–18. doi: 10.1096/fj.13-247635 (PMC4139899; doi:10.1096/fj.13-247635)
Supplement: Supplemental Data [file supp_fj.13-247635_13-247635SuppData.zip › Suppl.Fig. S4.pdf]

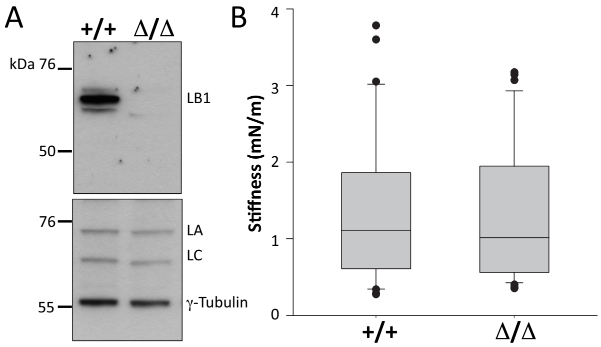

Figure S4 - LB1 deficiency did not affect nuclear stiffness in mouse embryonic fibroblasts (MEFs).

AFM nuclear stiffness analysis of wild type LB1+/+ (+/+) and LB1-null (Δ/Δ) MEFs. MEFs were serum-deprived for 3 days to induce a state of quiescence comparable to that of human ADLD fibroblasts (percentage BrdU-positive cells ± SEM Proliferating, 17.93 ± 3.28; quiescent, 6.00 ± 1.94, $p < 0.05$ Mann–Whitney Rank Sum Test). (*A*) Representative western blots of LB1, LA/C and γ-tubulin protein expression in +/+ and Δ/Δ MEF total lysates. (*B*) Box plot of average nuclear stiffness of +/+ and Δ/Δ MEFs. A total of 73 nuclei from +/+ (n = 30) and Δ/Δ (n = 43) MEFs were analyzed in 4 independent experimental sessions. $p = 0.97$, Mann–Whitney Rank Sum Test.
